# Dysregulation of junctional adhesion molecule‐A contributes to ethanol‐induced barrier disruption in intestinal epithelial cell monolayers

**DOI:** 10.14814/phy2.13541

**Published:** 2017-12-06

**Authors:** Daniel M. Chopyk, Pradeep Kumar, Reben Raeman, Yunshan Liu, Tekla Smith, Frank A. Anania

**Affiliations:** ^1^ Division of Digestive Diseases Department of Medicine Emory University School of Medicine Atlanta Georgia

**Keywords:** Junctional adhesion molecule‐A, ethanol, intestinal epithelial barrier, Caco‐2, Rap2, myosin light chain kinase

## Abstract

Alcohol consumption promotes loss of intestinal barrier function. However, mechanisms by which ethanol affects the tight junction (TJ), the cellular structure responsible for maintaining the gut epithelial barrier, are not well understood. Three classes of transmembrane proteins comprise TJs: occludin, claudins, and junctional adhesion molecules (JAMs). It has recently been postulated that JAM‐A (F11R), the most abundant JAM expressed in intestinal epithelium, regulates “leak” pathway flux, a paracellular route for the nonselective permeation of large solutes. Since transluminal flux of many gut‐derived antigens occurs through this pathway, we investigated the role of JAM‐A in ethanol‐induced disruption of the intestinal epithelial barrier. Using Caco‐2 and SK‐CO15 monolayers, we found that ethanol induced a dose‐ and time‐dependent decrease in JAM‐A protein expression to about 70% of baseline levels. Alcohol also reduced Ras‐related protein 2 (Rap2) activity, and enhanced myosin light chain kinase (MLCK) activity, changes consistent with impaired JAM‐A signaling. Stable overexpression and shRNA‐mediated knockdown of JAM‐A were employed to investigate the role of JAM‐A in paracellular‐mediated flux following alcohol exposure. The paracellular flux of 40‐kDa fluorescein isothiocynate (FITC)‐dextran following ethanol treatment was decreased by the overexpression of JAM‐A; conversely, flux was enhanced by JAM‐A knockdown. Thus, we conclude that ethanol‐mediated control of JAM‐A expression and function contributes to mechanisms by which this chemical induces intestinal epithelial leakiness.

## Introduction

The most recent World Health Organization data report that alcohol use accounts for approximately 3.3 million annual deaths globally, a large proportion of which are the result of alcoholic liver disease (ALD) (World Health Organization, 2014). Approximately 50% of deaths attributable to cirrhosis are the result of alcohol abuse (World Health Organization, 2014). Recently, it has become clear that alcohol exposure causes impaired intestinal barrier function (Keshavarzian et al. 1999; Patel et al. 2015), leading to elevated serum endotoxin (Bode et al. 1987; Fukui et al. 1991; Parlesak et al. 2000; Bala et al. 2014). Since blood from the intestines drains into the venous portal system, it is the liver that first encounters elevated levels of lipopolysaccharide (LPS) and other gut‐derived pathogen associated molecular patterns (PAMPs) (Patel et al. 2015; Szabo 2015). A growing body of evidence suggests that the resulting liver inflammatory response, primarily driven via LPS‐Toll‐Like Receptor‐4 (TLR‐4) interactions on liver‐resident Kupffer cells, promotes ALD pathogenesis (Wang et al. 2012; Nagy 2015).

Alcohol causes profound physiological changes to epithelial cells lining the gut (Patel et al. 2015). Of particular interest are the effects of ethanol on the tight junction (TJ), the structure responsible for the formation, maintenance, and regulation of epithelial paracellular barrier function (Farquhar and Palade 1963). TJs consist of three groups of transmembrane proteins: occludin, claudins, and junctional adhesion molecules (JAMs) (van Itallie and Anderson 2014). Although TJ proteins share some functional redundancy, each molecule has several unique roles (Raleigh et al. 2010; van Itallie and Anderson 2014; Lingaraju et al. 2015). For example, the claudin family of proteins has been demonstrated to regulate permeability to ions and small solutes in a charge selective manner (Koval 2015; Lingaraju et al. 2015). However, the individual functions of occludin and JAMs are much less understood.

Numerous studies have demonstrated that ethanol, its metabolites, and alterations of the gut microbiome suppress intestinal occludin expression (Chen et al. 2015; Mir et al. 2015; Samak et al. 2016). Moreover, occludin knockout (KO) mice were recently found to be more susceptible to ethanol‐induced gut barrier dysfunction and liver injury (Mir et al. 2015). However, the lack of a pronounced intestinal phenotype in occludin KO mice in the absence of alcohol suggests that this protein may play an accessory role in regulating epithelial barrier function (Saitou et al. 2000). Similarly, mice lacking JAM‐A (F11R), the prominent JAM expressed in the intestine, were demonstrated to have elevated serum endotoxin levels compared to wild‐type (WT) littermates after 8‐weeks of high‐fat diet (Rahman et al. 2016). This finding supports the proposed functional role that JAM‐A regulates paracellular permeability to macromolecular solutes via regulating the contractile tone of the apical cytoskeleton through modulation of myosin light chain kinase (MLCK) (Monteiro et al. 2013). Ethanol has been demonstrated to enhance MLCK activity at least in part via activation of the Ras homolog gene family member A (RhoA), another proposed signaling partner of JAM‐A (Monteiro et al. 2013; Tong et al. 2013a,b; Elamin et al. 2014).

Despite the demonstration of an important role of JAM‐A for TJ function, and that ethanol exposure interferes with molecules associated with JAM‐A signaling, the role of JAM‐A in ethanol‐induced gut barrier dysfunction has been left understudied.

Here, we hypothesized that dysfunction of JAM‐A contributes to ethanol‐induced epithelial barrier disruption and provide multiple approaches demonstrating that ethanol reduces JAM‐A protein expression, and disrupts its downstream signaling partners. Additionally, we provide evidence that directly associates JAM‐A expression with enhanced barrier function in the context of alcohol exposure.

## Materials and Methods

### Chemical reagents

Cell culture reagents including antibiotics were purchased from Corning (Corning, NY) and Sigma‐Aldrich (St. Louis, MO), except for fetal bovine serum which was purchased from Atlanta Biologicals (Flowery Branch, GA). Fluorescein isothiocynate (FITC)‐dextran 4‐kDa and 40‐kDa were purchased from Sigma‐Aldrich. Hanks Balanced Salt Solution with Ca^2+^/Mg^2+^ without phenol red (HBSS+) was purchased from Sigma‐Aldrich. Two hundred‐proof ethanol was purchased from Decon Labs (King of Prussia, PA) and was sterile‐filtered with Thermo Fisher Nalgene^TM^ bottle‐top vacuum filters (Waltham, MA).

### Antibodies

Mouse monoclonal antihuman JAM‐A was purchased from NovusBio (Littleton, CO; #H00050848‐M01). Goat polyclonal antihuman JAM‐A (#AF1033) and goat polyclonal antimouse JAM‐A (#AF1077) were both purchased from R&D Systems (Minneapolis, MN). Mouse monoclonal antioccludin, mouse monoclonal antizonula occludens‐2 (ZO‐2), and donkey polyclonal antigoat IgG (H+L)‐Alexa Fluor® 488 were purchased from Thermo Fisher (#33‐1500, #37‐4700, and #A‐11055). Mouse monoclonal anti‐*β*‐actin and mouse monoclonal antimyosin light chain kinase (MLCK) were purchased from Sigma‐Aldrich (#A5441 and #M7905). Mouse monoclonal anti‐FLAG (Sigma‐Aldrich #F1804) was a kind gift from Dr. Chris Yun (Emory University, Atlanta, GA). Goat antimouse IgG‐HRP was purchased from Santa Cruz (Dallas, TX; #sc‐2005).

### Cell culture

Caco‐2, SK‐CO15, and HEK293T cells were originally purchased from American Type Culture Collection (ATCC, Manassas, VA). Caco‐2 and SK‐CO15 cells were empirically pretreated for mycoplasma with plasmocin. All cells were maintained in DMEM supplemented with 10% FBS and 1% penicillin‐streptomycin, and 1 mmol/L sodium pyruvate additionally added for Caco‐2 cultures. Cells were split every 3–5 days.

### Caco‐2 cell transfection and generation of stable clones overexpressing JAM‐A

JAM‐A overexpression was achieved by transfection of Caco‐2 cells with a pCMV6‐F11R‐MycDDK plasmid purchased from OriGene (Rockville, MD; #RC221478). The plasmid was first expanded in NEB‐5*α* competent *E. Coli* purchased from New England BioLabs (Ipswich, MA; #C2988J) by heat shock transformation. Transformed bacteria were selected by overnight incubation at 37°C on Lysogeny Broth (LB) agar plates with 25 *μ*g/mL kanamycin (Sigma‐Aldrich). Selected colonies were expanded in 25 *μ*g/mL kanamycin LB broth incubated at 37°C and 250 rpm for 16‐h. Plasmid DNA was isolated using a QIAprep Spin Miniprep Kit (Qiagen; Hilden, Germany). The equivalent empty pCMV6 vector control plasmid (OriGene #PS100001) was previously expanded in a similar fashion.

One day before transfection, actively dividing Caco‐2 cells were seeded on a 6‐well plate in antibiotic‐free DMEM. Cells were 60–70% confluent the next morning, and media were replaced with Opti‐MEM™ reduced serum growth media (Thermo Fisher). The cells were then transfected with 3 *μ*g DNA of either plasmid, and 9 *μ*L Lipofectamine^®^ 2000 (Thermo Fisher) per well. Opti‐MEM™ was changed with complete growth media 6‐h posttransfection. Transfected cells were split 48‐h posttransfection, and were grown in increasingly higher concentrations of G418 up to ~700 *μ*g/mL (Sigma‐Aldrich) over several weeks. Selected clones were perpetually maintained in 700 *μ*g/mL G418 thereafter. Cells from passage 20–32 were used for transwell experiments.

### Generation of shRNA plasmid lentiviral vectors and Caco‐2 transduction

Bacterial stocks expressing plasmids encoding JAM‐A‐specific shRNA (Cat. #TRCN0000431967), pLKO.1 control shRNA, pLTR‐G envelope, and PCD/NL‐BH4 packaging plasmid were purchased from Sigma‐Aldrich. All bacterial stocks were expanded as described above using either 100 *μ*g/mL ampicillin (Sigma‐Aldrich) or 50 *μ*g/mL kanamycin (pLTR‐G only). Plasmid DNA was isolated as described above.

For generation of pseudoviral particles, actively dividing HEK293T cells were seeded in T75 flasks (Corning) in antibiotic‐free media and were cultured overnight. Subconfluent cultures (90%) were transfected with shRNA target (pLKO.1 or JAM‐A), helper plasmid (PCD/NL‐BH4), and envelope plasmid (pLTR‐G) DNA in an 8 *μ*g: 6 *μ*g: 4 *μ*g ratio using 30 *μ*L of lipofectamine^®^ LTX reagent and 30 *μ*L PLUS™ reagent (Thermo Fisher) in serum‐free DMEM. Viral supernatants were harvested 48‐h posttransfection and were cleared by centrifugation for 5‐min at 931*g* at 25°C and filtered through a 0.45 *μ*m pore PVDF syringe tip filter (Millipore; Billerax, MA). Viral stocks were aliquoted and frozen at −80°C until use.

For viral transduction, Caco‐2 cells at passage 30 were seeded on 6‐well plates and were grown to 100% confluence over 7‐day. On day 7, cells were incubated in a 1:1 dilution of media plus crude viral supernatant with polybrene (Santa Cruz) at a final concentration of 10 *μ*g/mL. Viral supernatants were replaced with fresh complete growth medium after 24‐h. After an additional 24‐h, the transduced cells were split onto new plates using media supplemented with 10 *μ*g/mL puromycin (Sigma‐Aldrich). Selection media were changed every 48‐h over 10‐d. The selected colonies present on day 10 were split into T75 flasks using 10 *μ*g/mL puromycin, and were continuously expanded and maintained under these conditions thereafter. Cells between passages 34–39 were used for transwell experiments.

### Transwell seeding and maintenance

Corning 24‐mm 6‐well and 6.5‐mm 24‐well 0.4 *μ*m pore polyester membrane transwell inserts were coated with 10 *μ*g/cm^2^ growth area rat tail collagen Type 1 (Sigma‐Aldrich) diluted in 70% ethanol. Transwells were left to dry under UV light overnight and were washed once with PBS and once with complete growth media. Actively dividing Caco‐2 or SK‐CO15 cells were split and seeded at an approximate density of 2 × 10^4^ cells/cm^2^ growth area (1 × 10^5^ cells per 6‐well insert, 6.67 × 10^3^ per 24‐well insert). Media were changed 6‐h after seeding, and every 48‐h thereafter. Experiments were carried out between days 15 and 20 after seeding (7–12 days postconfluence).

### Modified high‐density transwell seeding for transfected and transduced Caco‐2 experiments

Stable clones of transfected and transduced Caco‐2 cells exhibited greatly reduced growth rates compared to nontransformed cells due to the presence of selection antibiotics. To compensate, these cells were seeded on transwells at about 2.6 × 10^5^ cells/cm^2^ growth area (1.3 × 10^6^ cells per 6‐well insert, 8.5 × 10^4^ per 24‐well insert) to achieve confluence overnight. Media were changed 24‐h after seeding, and every 48‐h thereafter. Experiments were carried out between days 7 and 8.

### Ethanol treatment

Except for inserts used for FITC‐dextran and resistance studies, epithelial cell monolayers were prepared 24‐h in advance by individually separating monolayers into new 6‐well plates according to each condition (i.e., ethanol or control) with subsequent treatment with media to achieve 94% of the planned final volume. After overnight incubation at 37°C, monolayers were treated with media, sterile‐filtered 200‐proof ethanol (6% v/v), or a 1:1 mixed solution of both (3% v/v) to the final volume. These concentrations were based on previously published in vitro and in vivo studies (Ma et al. 1999; Tong et al. 2013b; Rubbens et al. 2016). All plates were sealed with parafilm to minimize the loss of ethanol as vapor during the treatment period (Rodriguez et al. 1992). It has been previously shown that sealing plates did not result in a significant in change in pH or cell viability within the prescribed treatment time frames (Rodriguez et al. 1992).

### TEER and FITC‐dextran flux

Only 6.5‐mm 24‐well transwell inserts were used for measuring transepithelial electrical resistance (TEER). Caco‐2 cells were washed twice with HBBS+, and were then incubated in fresh HBSS+ for 1‐h at 37°C. Next, 4‐kDa or 40‐kDa FITC‐dextran was pipetted into the apical compartment at a final concentration of 1 mg/mL, immediately followed by the addition of HBSS+, ethanol, or a 1:1 mixture of both, to both the apical and basal chambers (0, 3%, 6% v/v final concentrations of ethanol). Resistance was measured immediately after treatment (*t* = 0) and following 3‐h incubation using an EMD Millipore Millicell‐ERS Volt‐Ohm meter (Millipore; Model #MERSSTX01). Basal fluid fluorescence at 485 nm excitation and 525 nm emission was measured after the 3‐h incubation using a BioTek^®^ Synergy2 microplate reader with Gen5 software (Winooski, VT). Empty cell‐free collagen‐coated transwells were used to normalize monolayer resistances and record maximal FITC‐dextran flux. Only monolayers with minimum baseline TEER values consistent with confluence were used for analysis (≥250 Ω × cm^2^ for unmanipulated Caco‐2; ≥80 Ω × cm^2^ for pCMV6‐transfected Caco‐2). Inclusion of shRNA transduced Caco‐2 monolayers in data analysis was based solely on confluence by microscopic inspection.

### Protein lysate collection and western blot analysis

Epithelial cell monolayers were washed with ice‐cold PBS (Corning) twice and were lysed with ice‐cold RIPA lysis buffer (Alfa Aesar; Haverhill, MA) with cOmplete™ and PhosSTOP™ protease/phosphatase inhibitor cocktails (Roche; Basel, Switzerland). Lysates were sonicated and cleared by centrifugation at 9.3 k × *g* for 10‐min at 4°C. Protein quantifications were made by bicinchoninic acid assay (BCA assay; Thermo Fisher). Equal amounts of protein were then aliquoted in reducing LDS sample buffer using RIPA lysis buffer as diluent. Samples were reduced by heating at 95°C for 5‐ to 7‐min.

Unless otherwise stated, 20 *μ*g of protein was loaded in either NuPAGE™ 4–12% Bis‐Tris protein gels (Thermo Fisher) run in 3‐(N‐morpholino)propanesulfonic acid (MOPS) buffer (Thermo Fisher) or in 12% polyacrylamide gels made with Protogel® run in Tris‐Glycine SDS buffer (National Diagnostics; Atlanta, GA). All gels were transferred to PVDF membranes by electro‐transblotting. Blots were blocked with 5% nonfat milk in TBST for 1‐h at 25°C. All primary antibody incubations other than anti‐*β*‐actin (1:10,000 for 1‐h at 25°C) were overnight at 4°C at the following dilutions in 5% BSA TBST: antihuman JAM‐A (NovusBio) 1:500, antioccludin 1:1000, anti‐MLCK 1:10,000, anti‐ZO‐2 1:1000, antimouse JAM‐A 1:2000. Anti‐FLAG antibodies were used 1:1000 in 3% nonfat milk in TBST for overnight incubation at 4°C. Secondary antibody incubation was applied for 1‐h at 25°C using goat antimouse‐IgG‐HRP diluted to 1:5000 in 5% nonfat milk in TBST. All blots were developed using ECL solution made in‐house and autoradiographic imaging (VWR; Radnor, PA). Densitometry was measured using VisonWorks^®^LS UVP Image Acquisition and Analysis software version 8.1.2. Densitometry measurements for each target were normalized to respective *β*‐actin bands.

### Immunoprecipitation and kinase activity assay

Protein lysates were collected from Caco‐2 monolayers as described above except T‐per™ Tissue Protein Extraction Reagent (Thermo Fisher) was used to preserve enzymatic function. Lysates were quantified and snap‐frozen in liquid nitrogen on the day of harvest, and were thawed in water baths (25°C) on the day of immunoprecipitation (IP) and analysis. Smooth muscle/nonmuscle myosin light chain kinase (MLCK) was immunoprecipitated from 400 *μ*g of total protein lysate using magnetic SureBeads™ (Bio‐Rad; Hercules, CA) coated with 1 *μ*g mouse anti‐MLCK (Sigma‐Aldrich #M7905). IP was subsequently performed as outlined in Bio‐Rad's protocol. Kinase activity of the immunoprecipitated complexes was directly measured without elution using an ADP‐Glo™ kinase assay kit (Promega; Madison, WI) and synthetic peptide substrate MRCL3 (SignalChem; Richmond, British Columbia, Canada). Briefly, the MLCK‐SureBeads™ complexes were magnetized and all wash buffer was aspirated. The bead complexes were fully resuspended in 10 *μ*L of 1X kinase reaction buffer (40 mmol/L Tris‐HCl pH 7.5, 20 mmol/L MgCl_2_, 0.1 mg/mL BSA) and were divided into two‐5 uL aliquots each that were measured in duplicate. A 20 *μ*L mixture of MRCL3 peptide and ATP was added to the beads for a final concentration of 50 *μ*mol/L ATP and 0.4 *μ*g/mL MRCL3. The reaction mixtures were incubated at 25°C for 60‐min before the kinase reaction was stopped by addition of 25 *μ*L of ADP‐Glo™ reagent. The samples were incubated for another 40‐min at 25°C and then 50 *μ*L of kinase detection reagent was added. Luminescence was measured with the kinase‐Glo protocol on a GlowMax^®^ 20/20 Luminometer (Promega) after a final 30‐min incubation at 25°C. All sample measurements were adjusted for the luminescence of antibody‐coated SureBeads™ alone.

### Rap2 activation pulldown assay

Active Rap2 was analyzed via a RalGDS RBD Agarose bead pulldown assay kit according to the manufacturer's instructions (Cell BioLabs; San Diego, CA). Rap2 pulldown was conducted using 500–750 *μ*g of protein, and in some experiments lysates from Caco‐2 monolayers treated in duplicate were pooled. Western blots were performed as described above on the pulled down, active Rap2 and respective total protein samples using the respective antibodies at their recommended concentrations. Densitometric measurements for active Rap2 were normalized to the total Rap2/*β*‐actin ratio.

### LDH assay

Caco‐2 monolayers were washed twice with HBSS+ and were divided into separate, clean 6‐well plates according to planned treatment (0%, 3%, or 6% v/v ethanol). Cells were then incubated in HBSS+ at 94% of the planned final volume for 1‐h at 37°C, after which the remaining volume was added as HBSS+, 200‐proof ethanol, or a 1:1 mixture of both as described above. After 6‐h incubation at 37°C, apical solutions were collected and cleared by centrifuged at 9.3k × *g* for 10‐min at 4°C. Lactate dehydrogenase (LDH) activity was measured in triplicate using a cytotoxicity detection kit (Roche). Absorbance at 490 nm and 620 nm was measured using a BioTek^®^ Synergy2 microplate reader with Gen5 software. Results were normalized according to wells containing HBSS+ alone.

### Immunofluorescence confocal microscopy

Caco‐2 monolayers were washed with 0.05% Triton X‐100 (Thermo Fisher) in PBS (PBST) and were fixed by 20‐min RT incubation in 3.7% paraformaldehyde (Electron Microscopy Sciences; Hatfield, PA) in 0.05% PBST. Cells were then permeabilized with 0.1% PBST for 20‐min at RT followed by blocking with 5% BSA and 5% donkey serum in 0.05% PBST for 30‐min at RT. Monolayers were then excised from their inserts and were stained overnight at 4°C with goat antihuman JAM‐A (R&D Systems) 1:40 in 3% BSA diluted in 0.05% PBST. Secondary staining was done by 1‐h RT incubation in donkey antigoat IgG (H+L)‐Alexa Fluor® 488 1:1000 in 1% BSA diluted in 0.05% PBST. Samples were then mounted using Prolong™ Gold with DAPI (Thermo Fisher). Samples were visualized with an Olympus IX81 inverted microscope (Olympus Scientific Solutions Americas, Inc.; Waltham, MA).

Frozen sections of mouse ileum were thawed at RT for 10 min and were rehydrated with 0.05% PBST. Tissue was fixed with incubation in 100% methanol at −20°C for 10 min., followed by permeabilization in 0.3% PBST for 15 min at RT. Samples were blocked with 5% BSA and 5% donkey serum in 0.3% PBST for 1‐h at RT. Tissue was then stained overnight at 4°C with goat antimouse JAM‐A 1:100 in 1% BSA in 0.3% PBST. An additional tissue sample was incubated in 1% BSA in the absence of primary antibodies was included as a negative control. All samples were next stained with donkey antigoat IgG (H + L)‐Alexa Fluor® 488 1:1000 in 1% BSA diluted in 0.3% PBST for 1‐h at RT. Samples were mounted and imaged as described above.

### Mice and chronic plus binge ethanol in high‐fat diet model and in vivo assessment of gut permeability

All mouse work was outsourced to the Southern California Research Center for ALPD and Cirrhosis Animal and Morphology Core facilities. 8‐week‐old wild‐type male C57BL/6 mice were fed a modified high‐fat, high‐cholesterol Lieber‐DeCarli diet containing 1.26 g/kg cholesterol, 12.5% calories derived from milk‐fat, 16.7% calories derived from lard, and 8.4% calories derived from corn oil (Dyets, Inc.; Bethlehem, PA; Cat. #710383 and #710384). Mice were fed ethanol by an escalated dose (%v/v) of 1.45% for 3 day, 2.9% for 4 day, and 4.35% (~23.3% total calories). A cohort of control mice was pair‐fed an equivalent isocaloric diet without ethanol. Additionally, mice were given a once‐per‐week binge of ethanol starting at a dose of 3.5 g ethanol per kg body weight. The binge dosage was increased by an additional 0.5 g ethanol per kg body weight each consecutive week to a final dose of 6.0 g ethanol per kg body weight. Control mice were given an isocaloric glucose binge.

The sixth and final ethanol or glucose binge was conducted 1‐day prior to sacrifice. Prior to sacrifice, mice were additionally gavaged with 600 mg/kg 4‐kDa FITC‐dextran in PBS. Serum samples from each mouse were collected by facial vein bleeds 4‐h post FITC‐dextran gavage. Mice were then euthanized while under isoflurane anesthesia and serum was collected by cardiac puncture. Remaining blood was removed from livers prior to harvest by gentle perfusion with ice‐cold PBS by a butterfly needle inserted via the portal vein. Ileum samples were cleaned of associated fat, cut longitudinally, and gently washed in ice‐cold PBS to remove luminal contents. Short segments of distal ileum (~1 cm) were cut and flash frozen in liquid nitrogen for analysis by western blot. Remaining ileum segments were rolled using a flat toothpick and were frozen in Tissue‐Tek OCT compound (Sakura Finetek USA, Inc.; Torrance, CA) for sectioning.

### Histology

Liver tissue was fixed in 10% phosphate‐buffered formalin (Thermo Fisher) and embedded in paraffin. After sectioning, samples were stained with hematoxylin and eosin and were visualized using a Nikon® Eclipse E600 light microscope (Nikon Instruments, Inc.; Melville, NY).

### Statistical analyses

All data are reported as means ± SEM. Data were all analyzed using GraphPad Prism^®^ 7.0a software (GraphPad Software; La Jolla, CA). Differences among multiple groups were analyzed by one‐way ANOVA followed by Dunnett's multiple comparison test. Experiments involving only two treatment groups were analyzed by Student's *t*‐test. Statistical significance was set at *P <* 0.05 for all analyses.

## Results

### Ethanol induces a dose‐dependent reduction in Caco‐2 monolayer barrier function without affecting cell viability

**Figure 1:**
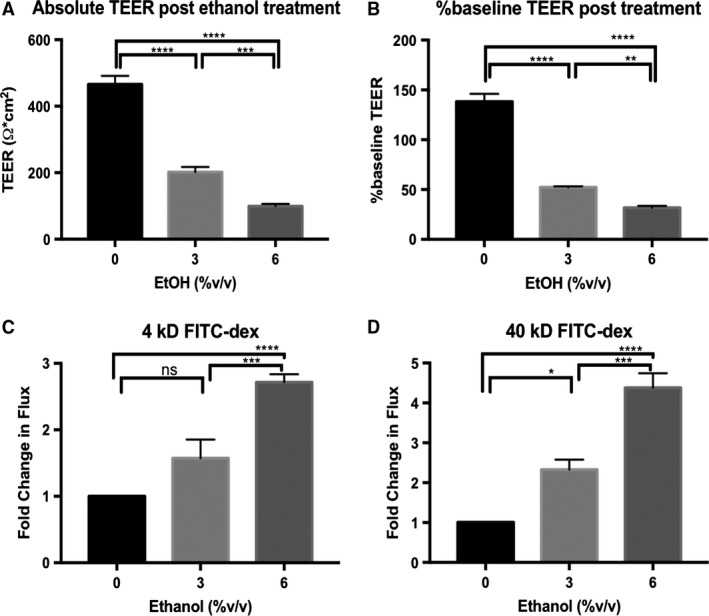
Ethanol induces a dose‐dependent reduction in transepithelial electrical resistance (TEER) associated with an increase in paracellular flux of FITC‐dextran in Caco‐2 monolayers. Caco‐2 monolayers were incubated in HBBS+ at 37°C for 1‐h. FITC‐dextran of either 4‐kDa or 40‐kDa in size was added to the apical compartments of the transwell plates at 1 mg/mL, followed by treatment of both apical and basal compartments with 0% (HBSS+ alone), 3%, or 6% v/v ethanol. TEER was measured immediately and again after 3‐h incubation. Fluorescence of the basal solutions following 3‐h incubation was measured and compared to solution collected from equivalently treated cell‐free transwell inserts. TEER readings are reported both as the normalized values after 3‐h incubation (A), and as the percent change from baseline (B). Flux of FITC‐dextran 4‐kDa (C), and 40‐kDa (D), were normalized to blank inserts and reported as fold‐change relative to monolayers treated with 0% ethanol. All data are representative means ± SEM of at least four individual monolayer inserts from two independent experiments. Data are analyzed by one‐way ANOVA, *P <* *0.05, **P <* *0.01, ***P <* *0.001, ****P <* *0.0001.

**Figure 2:**
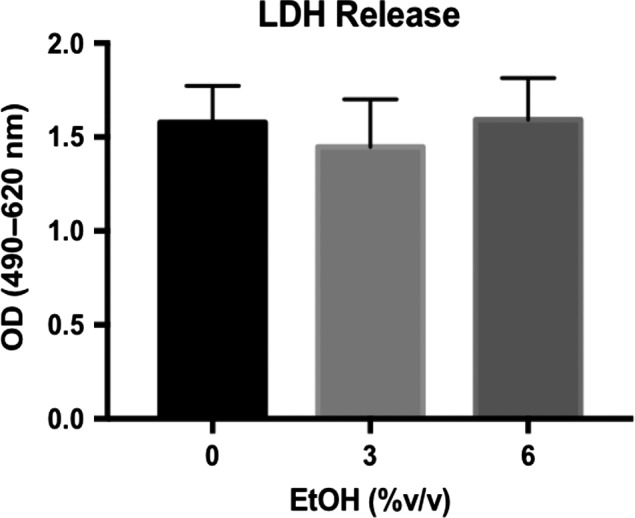
Ethanol up to 6% v/v does not increase Caco‐2 monolayer cell death. After incubating Caco‐2 monolayers in HBBS+ at 37°C for 1‐h, apical and basal compartments of the transwell plate were treated 0% (HBSS+ alone), 3%, or 6% v/v ethanol. LDH activity within apical extracellular media was measured after 6‐h incubation. Data are representative of means ± SEM of at least three individual monolayers from two independent experiments. Data are analyzed by one‐way ANOVA.

We employed the well‐established in vitro system of Caco‐2 monolayers to study the direct effects of ethanol on the intestinal epithelial TJ. Prolonged culture of this human colon adenocarcinoma cell line on transwell inserts results in their differentiation and permits development of mature TJs and brush border microvilli (Hidalgo et al. 1989; Ma et al. 1999). In agreement with previous studies, we observed a dose‐dependent reduction in monolayer TEER within 3‐h of ethanol treatment (Fig. 1A and B) (Ma et al. 1999; Tong et al. 2013b). Ethanol doses below ~1% v/v (~171 mmol/L) did not result in significant reductions in monolayer TEER (data not shown). Because TEER is only a measure of ionic flux, we also assessed the paracellular permeability of the monolayers using both small (4‐kDa) and large (40‐kDa) FITC‐dextran. Permeability of both sizes of FITC‐dextran was significantly increased by 6% ethanol exposure (Fig. 1C and D; 2.72 ± 0.29‐fold 6% vs. 0% 4‐kDa; 4.38 ± 0.36‐fold 6% vs. 0% 40‐kDa). Furthermore, ethanol dosages as high as 6% did not cause significant changes in cellular viability as measured by LDH release, even after 6‐h treatment (Fig. 2).

### Expression of JAM‐A, but not occludin or ZO‐2, was reduced in Caco‐2 and SK‐CO15 monolayers after prolonged ethanol exposure

**Figure 3:**
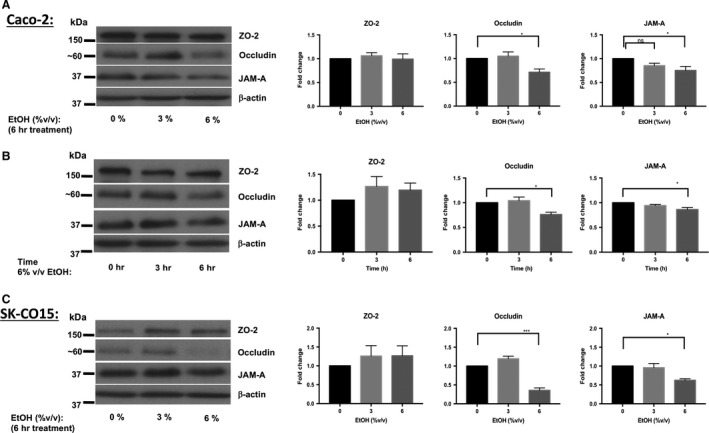
Ethanol induces a dose‐ and time‐dependent decrease in JAM‐A and occludin, but not ZO‐2, protein expression in Caco‐2 and SK‐CO15 monolayers. Caco‐2 monolayers 15‐day after seeding were incubated overnight followed by treatment with 0% (medium), 3%, or 6% v/v ethanol for an additional 6‐h incubation (A). Additional transwell plates were exposed to 6% ethanol in staggered fashion for 0‐ (medium alone), 3‐, or 6‐h (B). SK‐CO15 monolayers were also treated with 0%, 3%, or 6% v/v ethanol for 6 h (C) Total protein lysates were collected and analyzed by western blot. Images are representative of results of at least 6 (A), 4 (B), and 3 (C) independent experiments. Densitometry results are reported as the fold‐change in target protein expression (normalized to *β*‐actin) relative to monolayers treated with medium alone. Densitometry data are reported as means ± SEM and analyzed by one‐way ANOVA, *P <* *0.05, ***P <* *0.001.

**Figure 4:**
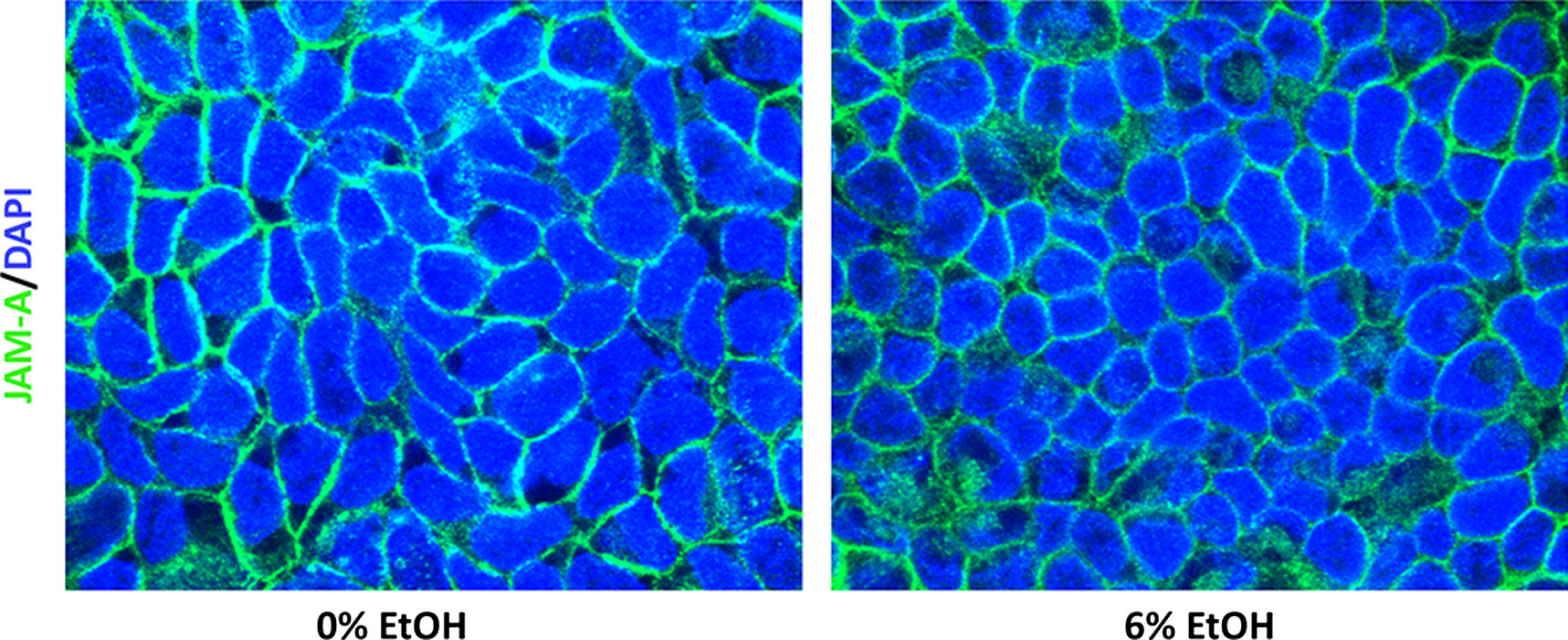
Subcellular localization of JAM‐A is not altered by ethanol exposure. Caco‐2 monolayers 15‐day after seeding were incubated overnight followed by treatment with 0% (medium) or 6% v/v ethanol for an additional 6‐h incubation. Monolayers were then fixed with paraformaldehyde, excised from their inserts, and stained with primary and fluorophore‐conjugated secondary antibodies. Images are representative of *n* = 4 monolayers per treatment.

The effect of ethanol on JAM‐A protein expression in Caco‐2 monolayers was investigated by dose–response in which cells were exposed to 3% or 6% ethanol for 6‐h. A significant reduction in JAM‐A protein expression compared to controls was observed after 6% ethanol treatment (Fig. 3A; 0.75 ± 0.08‐fold change 6% vs. 0%), whereas 3% ethanol exposure resulted in a nonsignificant trend toward reduced expression (*P = *0.16). Similar changes in occludin expression were also observed at both 3% and 6% ethanol, but no significant change was observed in ZO‐2 protein expression at either concentration (Fig. 3A). We also performed a time course of 6% ethanol treatment to examine the time frame of JAM‐A protein reduction in Caco‐2 monolayers. Our data demonstrate that prolonged exposure to ethanol for at least 6‐h was required to cause a significant reduction in JAM‐A and occludin protein expression (Fig. 3B). Consistent with dose–response analysis, we did not observe any significant change in ZO‐2 protein levels. We also found that at 9‐h JAM‐A protein expression was reduced but neither ZO‐2 nor occludin levels were reduced (data not shown). SK‐CO15 monolayers were also treated with ethanol to test further applicability of these findings beyond Caco‐2 cells. As with Caco‐2, protein expression of both JAM‐A and occludin, but not ZO‐2, was significantly reduced by 6% ethanol treatment for 6‐h (Fig. 3C; 0.62 ± 0.04‐fold change in JAM‐A 6% vs. 0%). We further hypothesized that the reduction in JAM‐A expression would be accompanied by disruption of its subcellular localization. Surprisingly, imaging of Caco‐2monolayers by confocal immunofluorescence microscopy revealed that JAM‐A localization was unaltered after 6% ethanol treatment for 6‐h (Fig. 4).

### Treatment of Caco‐2 monolayers with ethanol alters the activation status of JAM‐A signaling partners Rap2 and MLCK

**Figure 5:**
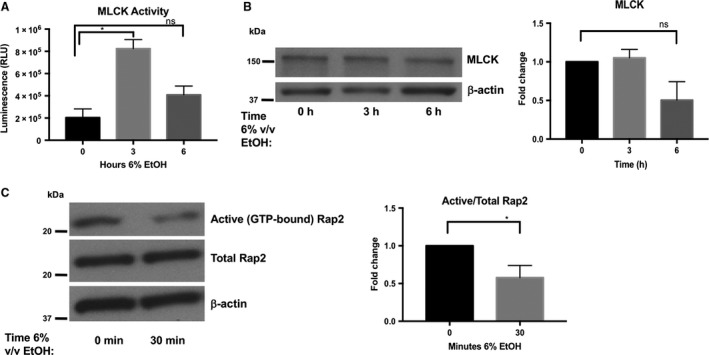
Ethanol exposure is associated with a transient increase in myosin light chain kinase (MLCK) phosphorylation activity as well as a decrease in Rap2 activation. Equal amounts of protein collected from Caco‐2 monolayers treated with 6% v/v ethanol for 0‐ (medium only), 3‐, or 6‐h, were incubated with antibody‐coated magnetic protein beads to immunoprecipitate MLCK over 1‐h. MLCK phosphorylation activity was measured directly on these bead complexes by Promega ADP‐Glo™ kinase assay kit using 50 *μ*mol/L ATP and 0.4 *μ*g/mL synthetic peptide substrate MRCL3. (A) MLCK phosphorylation activity in relative luminescence units (RLU) is reported as means ± SEM. (B) Total MLCK expression was not significantly different among samples; data representative of two independent experiments and was analyzed by one‐way ANOVA, *P <* *0.05. Rap2 activity was analyzed by pulldown of lysates collected from monolayers treated with 6% v/v ethanol for 0 or 30‐min (C). Rap2 western blot and densitometry are representative of three independent experiments. Data are analyzed by Student's *t*‐test, *P <* *0.05.

Because JAM‐A contributes to maintenance of intestinal epithelial barrier function through both structural and signaling functions (Monteiro et al. 2013), we hypothesized that ethanol exposure would result in perturbations of JAM‐A signaling partners MLCK and Rap2. Corroborating previously published observations (Ma et al. 1999; Tong et al. 2013b; Elamin et al. 2014), MLCK immunoprecipitation from Caco‐2 monolayer lysates followed by in vitro kinase activity analysis revealed a significant upregulation of MLCK activity within 3‐h of 6% ethanol treatment, which began to normalize within 6‐h (Fig. 5A). This increase in MLCK phosphorylation activity (~4‐fold) was observed without significant changes in total MLCK protein expression though there was a trend toward diminished MLCK expression (Fig. 5B). Interestingly, activation of the small GTPase Rap2, an upstream inhibitor of MLCK proposed to be regulated by JAM‐A (Monteiro et al. 2013), was significantly reduced by acute (30‐min) exposure to 6% ethanol (Fig. 5C; 0.58 ± 0.16‐fold 6% vs. 0%).

### Overexpression of JAM‐A in Caco‐2 monolayers is associated with increased TEER and protects against ethanol‐induced barrier dysfunction

**Figure 6:**
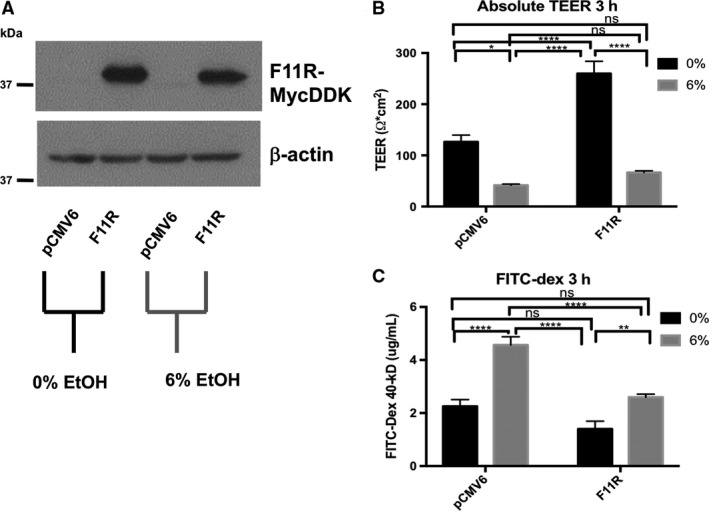
JAM‐A overexpression in Caco‐2 monolayers is associated with increased TEER and enhanced barrier function. Transfected Caco‐2 monolayers with stable expression of Myc‐FLAG tagged JAM‐A (F11R) or pCMV6 empty vector were incubated in HBBS+ at 37°C for 1‐h. FITC‐dextran 40‐kDa was added to the apical compartments of the transwell plates, followed by treatment of both apical and basal compartments with 0% (HBSS+ alone) or 6% v/v ethanol. TEER was measured immediately and again after 3‐h incubation. Expression of the tagged‐JAM‐A in the differentiated monolayers was confirmed by western blot (A). TEER values (B), and FITC‐dextran concentration (C), of basal solutions after 3‐h incubation are reported as means ± SEM. Western blot representative of two independent experiments. All other data are representative of multiple individual monolayer inserts from two independent experiments. Data are analyzed by two‐way ANOVA, *P <* *0.05, **P <* *0.01, ***P <* *0.001, ****P <* *0.0001.

Because ethanol exposure reduced JAM‐A protein expression and shifted the activation status of JAM‐A signaling partners toward states that promoted epithelial barrier disruption, we were interested in examining whether manipulating JAM‐A expression would result in alteration of monolayer response to ethanol. To address this question, we selected stable lines of Caco‐2 cells transfected with plasmids expressing a pCMV6 driven Myc‐DDK tagged JAM‐A (F11R) or empty vector. JAM‐A overexpression in the pCMV6‐F11R‐MycDDK, but not empty vector, transfected cells was confirmed by anti‐FLAG western blot (Fig. 6A). Compared to controls, cells overexpressing the Myc‐DDK tagged JAM‐A formed monolayers with a near twofold increase in resistance in the absence of alcohol (Fig. 6B, 126.4 ± 13.09 vs. 260 ± 23.95 Ω × cm^2^). Treatment of monolayers overexpressing JAM‐A with 6% was associated with a significant reduction in resistance versus untreated cells (Fig. 6B). There was no significant difference in monolayer TEER between empty vector and F11R‐MycDDK transfected Caco‐2 monolayers following 6% ethanol treatment (Fig. 6B, 41.66 ± 2.12 vs. 66.55 ± 3.37 Ω × cm^2^). Surprisingly, the resistance of Caco‐2 monolayers overexpressing MycDDK‐tagged JAM‐A that were treated with ethanol also did not significantly differ from cells transfected with the empty pCMV6 vector that were not exposed to ethanol (Fig. 6B). Therefore, to better understand if JAM‐A overexpression might protect against ethanol‐induced epithelial barrier loss, we assessed the paracellular permeability of the transfected Caco‐2 monolayers using 40‐kDa FITC‐dextran as in Figure 1D. We calculated the FITC‐dextran concentration of the basal solution 3‐h post 6% ethanol treatment by standard curve for more detailed analysis. In the absence of alcohol, the overexpression of JAM‐A caused nonsignificant trend toward reduced monolayer permeability (Fig. 6C, 2.25 ± 0.26 vs. 1.40 ± 0.30 *μ*g/mL). However, 40‐kDa FITC‐dextran permeability following ethanol treatment was significantly reduced by about 50% in monolayers overexpressing JAM‐A compared to empty‐vector transfected cells (Fig. 6C, 4.57 ± 0.31 vs. 2.60 ± 0.12 *μ*g/mL). Interestingly, the FITC‐dextran permeability of ethanol‐treated pCMV6‐F11R‐MycDDK transfected Caco‐2 monolayers did not significantly differ from empty‐vector monolayers that were not treated with ethanol. Together, these TEER and FITC‐dextran observations suggest that the overexpression of JAM‐A in Caco‐2 monolayers was associated with a “tighter” barrier even after ethanol exposure.

### Knockdown of JAM‐A in Caco‐2 monolayers is associated with exacerbated disruption of barrier function induced by ethanol exposure

**Figure 7:**
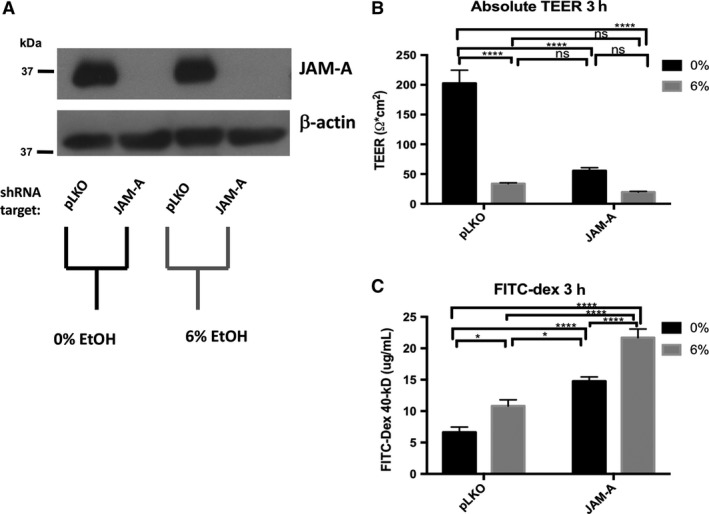
JAM‐A deficiency is associated with decreased Caco‐2 monolayer TEER and enhanced paracellular permeability. Transduced Caco‐2 monolayers with either JAM‐A specific shRNA or scrambled control (pLKO) were incubated in HBBS+ at 37°C for 1‐h. FITC‐dextran 40‐kDa was then added to the apical compartments of the transwell plates, followed by treatment of both apical and basal compartments with 0% (HBSS+ alone) or 6% v/v ethanol. TEER was measured immediately and again after 3‐h incubation. Knockdown of JAM‐A in the differentiated monolayers was confirmed by western blot (A). TEER values (B), and FITC‐dextran concentration (C), of basal solutions after 3‐h incubation are reported as means ± SEM. Western blot representative of two independent experiments. All other data are representative of multiple individual monolayer inserts from three independent experiments. Data are analyzed by two‐way ANOVA, *P <* *0.05, **P <* *0.01, ***P <* *0.001, ****P <* *0.0001.

Since our observations suggested that overexpression of JAM‐A serves to protect against ethanol‐induced barrier loss we sought to test the converse hypothesis, that is, whether JAM‐A knockdown would result in impaired barrier function. To address this question, we employed shRNA‐mediated knockdown of JAM‐A. Western blots performed on 7‐day‐old monolayers from pLKO and JAM‐A shRNA transduced Caco‐2 cells revealed virtually complete knockdown of JAM‐A protein levels (Fig. 7A). Knockdown of JAM‐A caused a dramatic reduction in Caco‐2 monolayer TEER (Fig 7B, 202.62 ± 22.17 vs. 55.46 ± 5.29 Ω × cm^2^). Furthermore, TEER of monolayers expressing JAM‐A targeted shRNA was not significantly reduced by ethanol, nor did their resistance significantly differ from pLKO‐transduced monolayers that were treated with ethanol. Paracellular permeability of 40‐kDa FITC‐dextran was also dramatically increased by JAM‐A knockdown (Fig. 7C). FITC‐dextran concentrations collected from ethanol‐naïve monolayers with JAM‐A knockdown were significantly greater than those collected from ethanol‐treated pLKO‐transduced Caco‐2 (Fig. 7C, 14.75 ± 0.70 vs. 10.82 ± 0.98 *μ*g/mL). Ethanol treatment significantly enhanced the paracellular permeability of JAM‐A shRNA transduced Caco‐2 monolayers to FITC‐dextran despite the relatively modest effects ethanol treatment had on TEER in these cells (Fig. 7C, 14.75 ± 0.70 vs. 21.71 ± 1.36 *μ*g/mL). Together, these results suggest that JAM‐A plays a pivotal role in maintenance of intestinal epithelial barrier function as measured by both electrical resistance and paracellular permeability to inert solutes.

### Chronic plus binge ethanol disrupts subcellular localization of JAM‐A, but not its total expression, in the ileum of high‐fat, high‐cholesterol Lieber‐DeCarli diet fed mice

**Figure 8:**
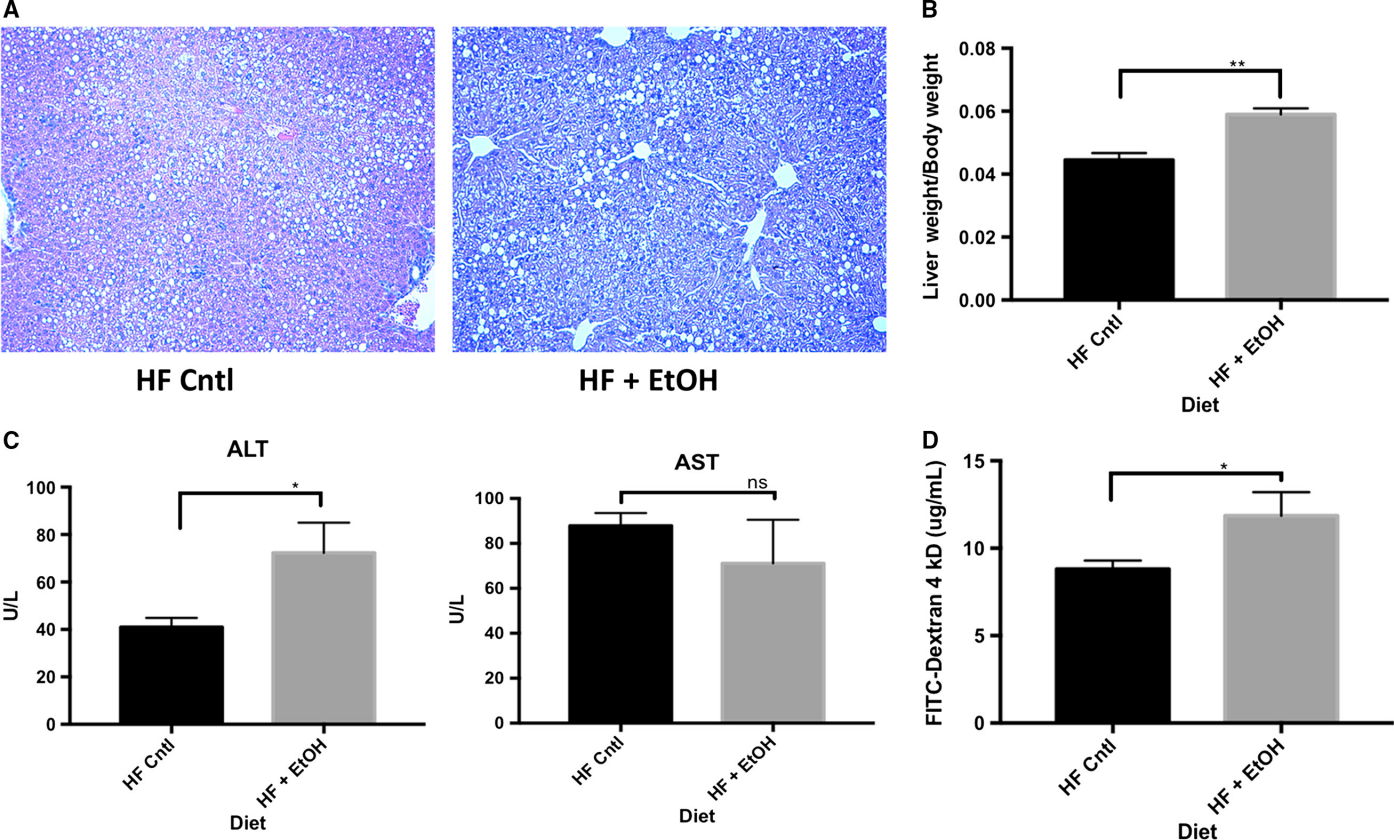
Chronic plus binge ethanol feeding exacerbates liver injury and increases intestinal permeability in mice fed a high‐fat, high‐cholesterol liquid diet. 8‐week‐old wild‐type C57BL/6 mice were fed a modified high‐fat, high‐cholesterol Lieber‐DeCarli diet with or without ethanol plus once per week ethanol or glucose binge for 6‐weeks. Liver injury was assessed by comparing hematoxylin and eosin stained liver sections (A), liver to body weight ratios (B) and serum AST and ALT levels (C). Additionally, prior to sacrifice, intestinal permeability was assessed by measuring serum concentration of 4‐kDa FITC‐dextran. Mice were gavaged with 600 mg/kg 4‐kDa FITC‐dextran and serum samples were collected by cheek bleed after 4‐h. All data are representative of *n* = 4 ethanol‐fed mice and *n* = 5 pair‐fed controls. Data are analyzed by students *t*‐test, *P <* *0.05, **P <* *0.01.

**Figure 9:**
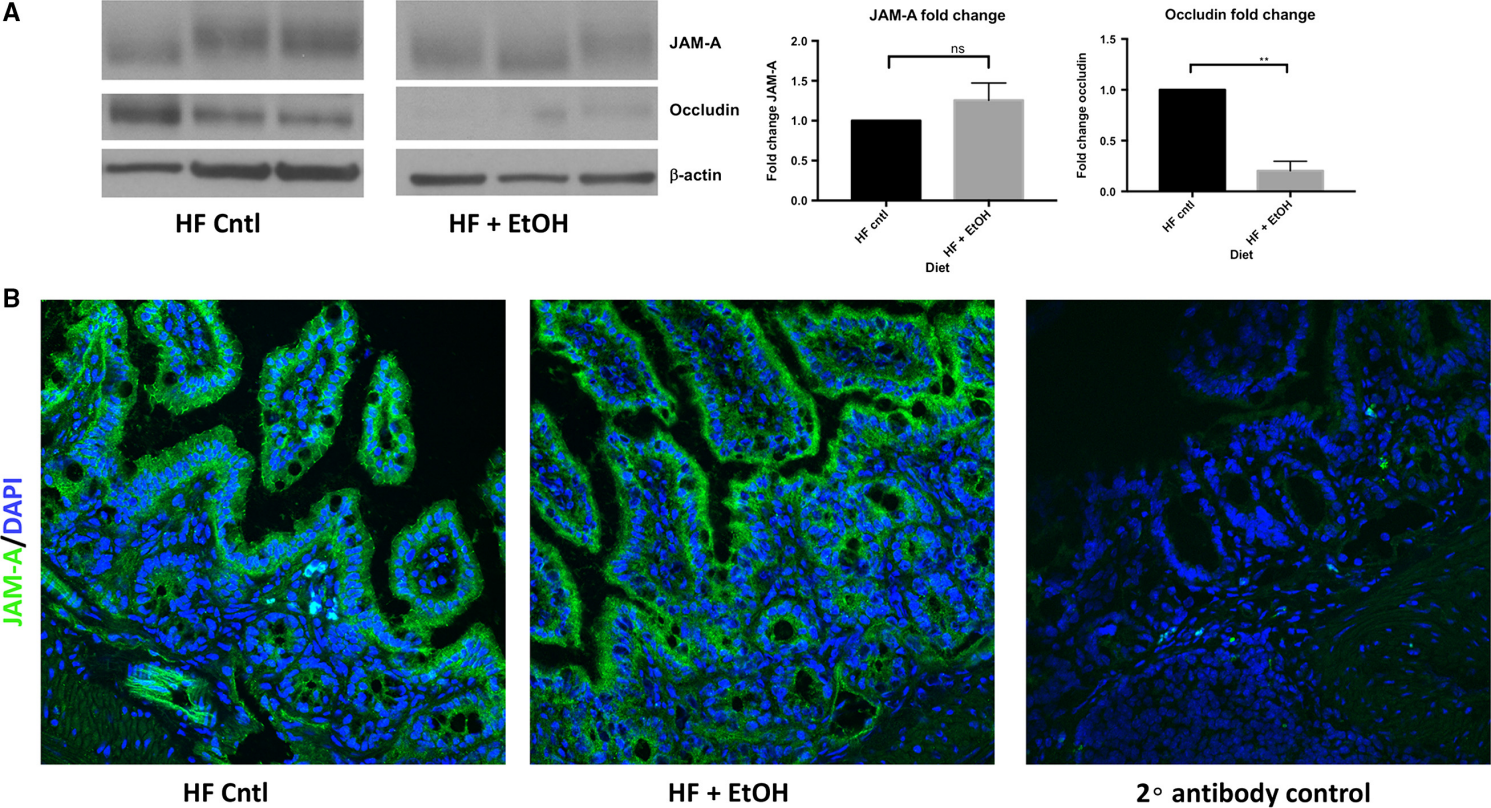
Chronic plus binge ethanol feeding disrupts JAM‐A subcellular localization, but not its overall protein expression, in the ileum high‐fat, high‐cholesterol liquid diet fed mice. 8‐week‐old wild‐type C57BL/6 mice were fed a modified high‐fat, high‐cholesterol Lieber‐DeCarli diet with or without ethanol plus once per week ethanol or glucose binge for 6‐weeks. Total protein lysates (30 *μ*g per sample) of distal ileum tissue were analyzed by western blot. Depicted image is from nonadjacent wells ran on the same gel (A). Densitometry data are representative of *n* = 3 mice per group and was analyzed by Student's *t*‐test, **P <* *0.01. Additionally, subcellular localization of JAM‐A within ileum was assessed by confocal immunofluorescence microscopy (B). Immunofluorescence images are representative of *n* = 4 ethanol‐fed mice and *n* = 5 pair‐fed controls.

In order to relate our in vitro observations to an in vivo model of ethanol feeding, we employed the services of Southern California Research Center for ALPD and Cirrhosis Animal and Morphology Core. Through these services, 8‐week‐old C57BL/6 WT mice were subjected to 6‐weeks of a modified high‐fat, high‐cholesterol Lieber‐DeCarli diet plus once per week ethanol binge. Ethanol‐fed mice suffered greater liver injury as measured by histologic assessment, liver/body weight ratio, and elevated serum ALT (Fig. 8A‐C). Prior to sacrifice, mice were gavaged with a 4‐kDa FITC‐dextran solution to measure intestinal epithelial permeability as described previously (Rahman et al. 2016). As shown in Figure 8D, serum FITC‐dextran concentration was significantly higher in the ethanol‐fed mice, suggesting that ethanol increased intestinal epithelial permeability of these animals. Although ileum occludin expression was dramatically reduced in ethanol‐fed mice, we observed no effect on total protein expression of JAM‐A (Fig. 9A). Because many cell types other than enterocytes express JAM‐A, such as endothelial cells and leukocytes (Luissint et al. 2014), we employed confocal immunofluorescence imaging to visualize JAM‐A expression in the intestinal mucosa. Our data demonstrate relocalization of JAM‐A from the junctions between enteroctyes of ethanol‐fed mice (Fig. 9B).

## Discussion

In this study, Caco‐2 monolayers were used to reaffirm past observations that ethanol causes a significant disruption of monolayer barrier function (Ma et al. 1999; Tong et al. 2013b). Enhanced permeability of relatively large 40‐kDa FITC‐dextran particles reported here supports the hypothesis that “leak” pathway regulators such as JAM‐A are involved in the mechanisms by which ethanol disrupts the intestinal epithelial barrier, and corroborates observations from other published studies (Mitzscherling et al. 2009; Yu et al. 2013; Thomes et al. 2015). By western blot we observed a consistent reduction in JAM‐A expression with no alteration in its subcellular localization following ethanol exposure in both a dose‐ and time‐dependent manner in vitro. This observation was apparently a consequence of prolonged exposure to ethanol, as the reduction in JAM‐A protein levels was not observed until at least 6‐h of treatment. We observed similar reductions in occludin protein levels, which corroborates previously published observations, as well as our in vivo results reported here (Tong et al. 2013b; Elamin et al. 2014; Chen et al. 2015; Mir et al. 2015; Samak et al. 2016). Surprisingly, in contrast to our Caco‐2 observations, JAM‐A expression in the ileum was unaltered in chronic plus binge ethanol‐fed mice. Instead, possible JAM‐A dysfunction was noted by disruption of its subcellular localization of JAM‐A in enterocytes. This discrepancy might be explained by the metabolism of ethanol to acetaldehyde and other metabolites that occurs in vivo, but not in Caco‐2 cells, which lack the expression of alcohol dehydrogenase (Koivisto and Salaspuro 1997). Moreover, mice in this study were sacrificed ~24‐h post final ethanol gavage, while it has been noted that the maximal amount of liver damage following a single ethanol binge occurs at ~9‐h (Ki et al. 2010). Therefore, it is a possible that total intestinal JAM‐A protein content might change in a similar time frame, and thus JAM‐A expression, but not its localization, may recover within 24‐h acute ethanol binge. We also found that ethanol in vitro did not result in significant changes in the expression of ZO‐2, a TJ scaffolding protein that associates with JAM‐A (Monteiro et al. 2013). Therefore, it is likely that the observations we have made are due to a direct regulatory effect of ethanol or acetaldehyde on JAM‐A, rather than a secondary effect caused by disruption of the other complexed partner molecules.

**Figure 10:**
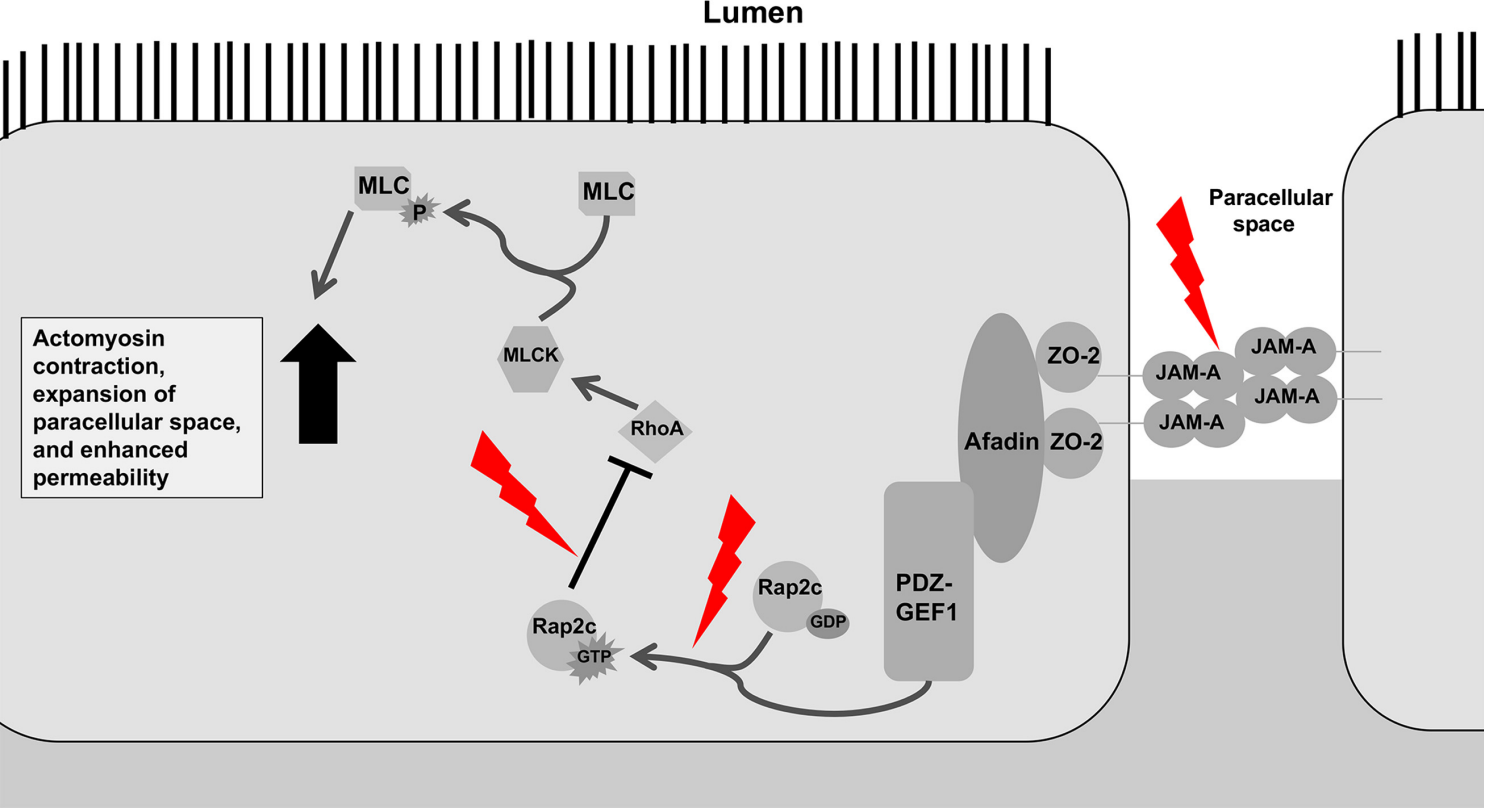
JAM‐A Barrier Promoting Signaling Pathway and Perturbations Induced by Ethanol. JAM‐A associates directly with ZO‐2, which forms a complex with Afadin and the guanine‐nucleotide exchange factor PDZ‐GEF1. This complex activates Rap2c by promoting GTP binding. Active Rap2c inhibits RhoA‐mediated activation of MLCK and downstream MLC phosphorylation which promotes barrier function. Ethanol exposure disrupts upstream targets, disinhibiting RhoA, and resulting in enhanced MLC phosphorylation. Contraction of the apical actomyosin ring follows, leading to an expanded paracellular space and increased solute flux. Perturbations induced by ethanol depicted as red lightning bolts. Arrows indicate activation, whereas bar‐headed lines indicate inhibition.

Although JAM‐A protein expression in epithelial monolayers was reduced to about 70% of baseline levels, observed after 6‐h ethanol treatment, reductions in monolayer TEER occurred within an acute time frame, 3 h. Therefore, we were interested in whether JAM‐A signaling dysfunction may precede alterations in its expression. Monteiro et al. recently suggested that JAM‐A contributes to intestinal epithelial barrier function through regulation of MLC phosphorylation via the Rap2c‐RhoA‐MLCK signaling axis, in Figure 10 (Monteiro et al. 2013). Hence, in the absence of JAM‐A, MLC phosphorylation increases leading to apical actomyosin contraction and expansion of the paracellular space, resulting in a leakier monolayer (Monteiro et al. 2013). Here we reported that ethanol exposure induced a significant increase in MLCK phosphorylation activity within 3‐h of ethanol treatment. Our findings corroborate previous in vitro and in vivo observations that ethanol enhances phosphorylation of MLC in intestinal epithelial cells (Ma et al. 1999; Tong et al. 2013a,b; Elamin et al. 2014; Chen et al. 2015). Importantly, two of these studies performed by independent groups linked MLCK activation to upstream activation of RhoA (Tong et al. 2013a; Elamin et al. 2014). Although inducible nitric oxide synthase (iNOS) was proposed as one potential regulator of RhoA (Tong et al. 2013a), we questioned whether ethanol‐induced perturbations in Rap2 would also contribute to RhoA regulation. In this study, we provide the first evidence that ethanol causes an acute reduction in Rap2 activation. Therefore, we propose a novel paradigm by which ethanol induces a reduction in active Rap2, which contributes to activation of RhoA, and ultimately leads to enhanced MLCK activity and MLC phosphorylation. While additional studies will be necessary to solidify this hypothesis, it is tempting to speculate the possibility that inhibition of Rap2 is mediated through ethanol‐mediated disruption of JAM‐A signaling function (Fig. 10).

We also found that JAM‐A overexpression nearly doubled Caco‐2 monolayer TEER. However, this difference was abolished by ethanol treatment. In contrast, monolayers overexpressing JAM‐A displayed significantly less permeability to 40‐kDa FITC‐dextran in the presence, but not the absence, of alcohol. Together these parallel observations suggest several possible conclusions, all of which raise interesting questions for future studies: (1) Although they are often associated together, epithelial TEER and paracellular permeability to inert solutes might be under distinct, sometimes divergent mechanisms of regulation; (2) JAM‐A contributes to monolayer TEER, but may play a minimal role in the mechanisms by which ethanol reduces TEER; and (3) the greater baseline TEER of monolayers overexpressing JAM‐A indicated a sufficiently “tighter” barrier that was maintained throughout enough of the 3‐h treatment duration to result in a lower net flux of FITC‐dextran. While many of these questions need be addressed by future studies, near complete knockdown of JAM‐A alone abolished monolayer TEER to levels similar to those of ethanol‐treated controls. However, since ethanol exposure clearly does not induce profound downregulation of JAM‐A protein, the direct contribution of JAM‐A, independent of other TJ structural members, to the regulation of epithelial TEER remains unclear. Nevertheless, the dramatic enhancement of FITC‐dextran flux across Caco‐2 monolayers that results as a consequence of JAM‐A deficiency, both in the presence and absence of alcohol, provides solid evidence for the critical role that JAM‐A plays in regulating paracellular permeability.

In conclusion, we demonstrated that prolonged exposure of intestinal epithelial cells to ethanol diminishes JAM‐A protein expression in vitro, and potentially disrupts its subcellular localization in vivo. Acute exposure of Caco‐2 cells to alcohol was associated with perturbations of Rap2 and MLCK activity, signaling molecules regulated downstream of JAM‐A. Thus, our data raise the possibility that JAM‐A signaling dysfunction and reduction in its protein content contributes to ethanol‐induced intestinal epithelial barrier loss. Through both knockdown and overexpression studies, we demonstrate a critical role of JAM‐A in the regulation of paracellular permeability both in the absence and presence of ethanol. However, further studies will be required to discriminate any potentially divergent regulatory mechanisms of monolayer TEER versus permeability to solutes, and to further elucidate the effects that ethanol consumption has on JAM‐A in vivo and the timing at which they occur.

## Conflict of Interest

The authors have no conflicts of interest.
